# Metastatic Prostate Cancer to the Left Temporal Bone: A Case Report and Review of the Literature

**DOI:** 10.1155/2015/250312

**Published:** 2015-07-30

**Authors:** Erynne A. Faucett, Hal Richins, Rihan Khan, Abraham Jacob

**Affiliations:** ^1^Department of Otolaryngology–Head and Neck Surgery, University of Arizona College of Medicine, Tucson, AZ, USA; ^2^University of Arizona College of Medicine, Tucson, AZ, USA; ^3^Department of Medical Imaging, University of Arizona College of Medicine, Tucson, AZ, USA; ^4^University of Arizona Cancer Center, University of Arizona Ear Institute and University of Arizona Bio5 Institute, Tucson, AZ 85724, USA

## Abstract

Breast, lung, and prostate cancers are the three most common malignancies to metastasize to the temporal bone. Still, metastatic prostate cancer of the temporal bone is a rare finding, with approximately 21 cases reported in the literature and only 2 cases discovered more than 10 years after initial treatment of the primary. This disease may be asymptomatic and discovered incidentally; however, hearing loss, otalgia, cranial nerve palsies, and visual changes can all be presenting symptoms. We present the case of a 95-year-old man with history of primary prostate cancer treated 12 years earlier that was seen for new-onset asymmetric hearing loss and otalgia. The tympanic membranes and middle ears were normal; however, based on radiologic findings and eventual biopsy, the patient was diagnosed with extensive metastatic prostate cancer to the left temporal bone. This case (1) demonstrates that a high index of suspicion for unusual etiologies of seemingly benign symptoms must be maintained in elderly patients having prior history of cancer and (2) substantiates the value of temporal bone imaging when diagnosis may be unclear from history and physical exam.

## 1. Introduction

Prostate cancer is the most common cancer diagnosed in American men [1]. Approximately 233,000 new cases occur annually [2]. The highest incidence (60–70%) of prostate cancer is seen in men who are in their seventh decade of life [3]. In addition to age, other risk factors include African American race, family history, and diets high in protein and fats and low in fruits and vegetables [4, 5]. Patients are typically asymptomatic due to early detection by prostate-specific antigen (PSA) testing and digital rectal exams (DRE) but may present with outflow obstruction, hematuria, lower leg edema, and bone pain [1]. The 5-year survival rate for patients diagnosed with early-stage, local prostate cancer is almost 100% [2, 6]. Those patients diagnosed with late-stage, metastatic prostate cancer have a 5-year survival rate of only 28–30% [3, 6, 7]. Prostate cancer metastasizes in only a small number of patients and typically involves the bones, with the skull being the sixth most common bone affected [2, 7, 8].

The temporal bone, with rich blood supply and sluggish blood flow, provides a hospitable environment for hematogenous seeding of tumor cells [9–12]. Still, involvement of the temporal bone in metastatic adenocarcinoma is rare. In 1986, Kobayashi et al. performed a review of the world literature revealing 9 cases of prostatic metastases to the temporal bone [13]. Since that time, only 12 additional cases have been reported in the literature (Table 1). Patients with metastatic cancer of the temporal bone may be asymptomatic [14–16]. However, hearing loss, facial palsy, and/or signs similar to mastoiditis (e.g., otalgia, ear drainage, and vertigo) can be seen [17–19]. Due to the low incidence of cases of metastatic prostate cancer, the infrequent involvement in the skull, and the nonspecific symptoms patients present with, metastatic prostate cancer of the temporal bone may be difficult to diagnose. Diagnosis relies on appropriate imaging studies and eventual biopsy for histologic and immunohistochemical staining [5]. Treatment in these patients is primarily palliative and may include surgery, chemotherapy, and/or radiation [6, 20].

To date, only 2 cases of metastatic prostate cancer to the temporal bone presenting >10 years after treatment of the primary tumor have been reported in the literature. Here, we present the 3rd such case and discuss implications for workup.

## 2. Case Report

A remarkably alert and functional 95-year-old man with long standing history of bilateral, symmetric, age-related hearing loss developed new-onset asymmetric hearing loss in the left ear along with sharp unilateral ear pain. The pain was intermittent but sharp and intense. He did not have vertigo, tinnitus, aural pressure, drainage, or facial weakness. Past medical history was significant for prostate cancer diagnosed in 2002 (12 years prior to presentation) and treated with neoadjuvant androgen deprivation therapy (ADT) as well as radioactive seed implant. Other medical problems included Parkinson's disease, hypertension, diabetes mellitus type II, and gout.

The patient was initially seen at an outside hospital where his symptoms were attributed to Eustachian tube dysfunction or perhaps temporomandibular joint arthritis. He was prescribed ciprodex otic drops, Flonase, and Tylenol. An MRI of the brain with internal auditory canal protocol was offered for the asymmetric and presumed-sudden hearing loss; however, the patient declined. He was seen in our otology practice two weeks later for a second opinion. By that point, the ear pain had resolved but asymmetric hearing loss persisted. On physical exam, the ear canals, tympanic membranes, and middle ears were within normal limits. An audiogram showed bilateral sensorineural hearing loss with poorer threshold in the left ear than the right (Speech Recognition Threshold: right ear (RE) 40 dB HL, left ear (LE) 55 dB HL; Speech Discrimination: RE 88% 85 dB HL, LE 80% 90 dB HL). There was a significant decrease in thresholds seen in a previous audiogram performed in 2012 (Speech Recognition Threshold: RE 35 dB HL, LE 35 dB HL; Speech Discrimination: RE 96% 70 dB HL, LE 84% 70 dB HL). Steroid treatment for the hearing loss was discussed; however, the patient declined stating he did not want to take on the risks of high-dose steroids at his advanced age. Review of laboratory testing found PSA values (0–2.5 ng/mL) of 4.22, 4.82, and 6.43 for the years 2012, 2013, and 2014 respectively. Alkaline phosphatase was 92 (40–150) and lactate dehydrogenase was 204 (125–243).

**Figure 1:**
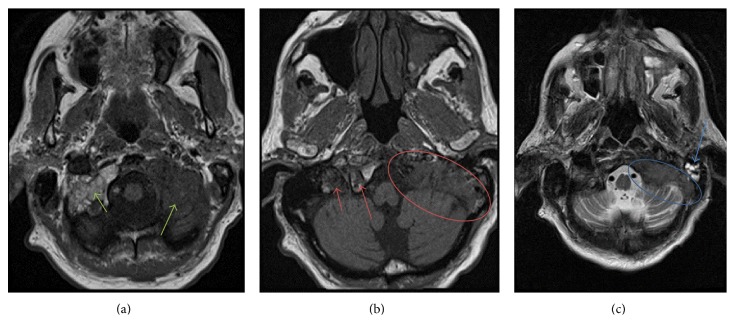
MRI scan of the head, 7/9/2014. (a) Axial T1 image shows a large posterior skull base mass. Note the normal bright fatty marrow (short green arrow) compared to darker signal from the mass (long green arrow). (b) Axial T1 image shows the mass (red oval) involving both the jugular foramen and the hypoglossal canal. Note the normal position of the contralateral jugular foramen (short red arrow) and the hypoglossal canal (long red arrow) for reference. (c) Axial T2 image shows involvement of the petrous temporal bone (blue oval) extending into the posterior inferior mastoid air cells, with bright reactive mastoid fluid (blue arrow).

**Figure 2:**
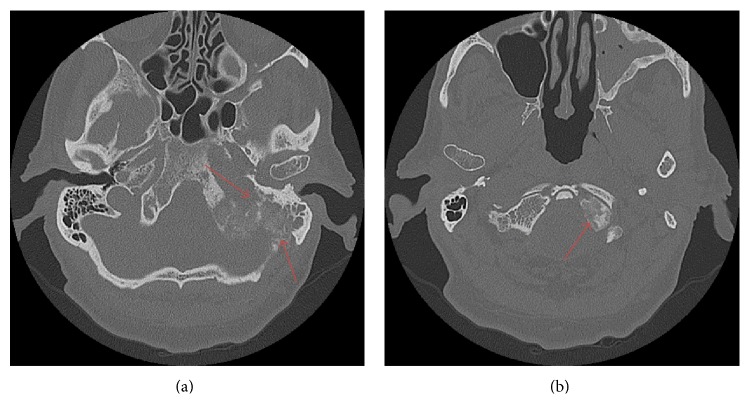
CT scan of the head without contrast, axial images, 8/11/2014, showing a destructive lesion centered within the left occipital bone with extension into the inferior mastoid temporal bone (a) and occipital condyle (b).

The patient was hesitant about obtaining an MRI because his ear pain had resolved; however, due to persistent asymmetry in hearing and prior history of cancer, we encouraged him to proceed with the imaging study as a precaution. The MRI revealed an extensive tumor involving the skull base, including the clivus and petrous temporal bone with extension into the posterior-inferior mastoid air cells (Figure 1). The tumor also bordered the posterior and medial portion of the foramen lacerum, obliterated the left jugular foramen, and involved the hypoglossal canal. A fine-cut, CT scan of the temporal bones was performed to evaluate extent of bony involvement and for operative planning. This confirmed presence of an infiltrative bony lesion involving the skull base (Figure 2).

Differential diagnosis included metastatic multiple myeloma, glomus tumor, and (most likely) metastatic disease. The patient's primary care physician recommended a bone marrow biopsy to the patient as a first step towards diagnosis because she felt that this was less invasive than temporal bone surgery and would rule out multiple myeloma. Bone marrow biopsy and laboratory panel were negative for multiple myeloma; therefore, the patient was taken to the operating room for left posterior petrosectomy and debulking/biopsy of his temporal bone lesion.

**Figure 3:**
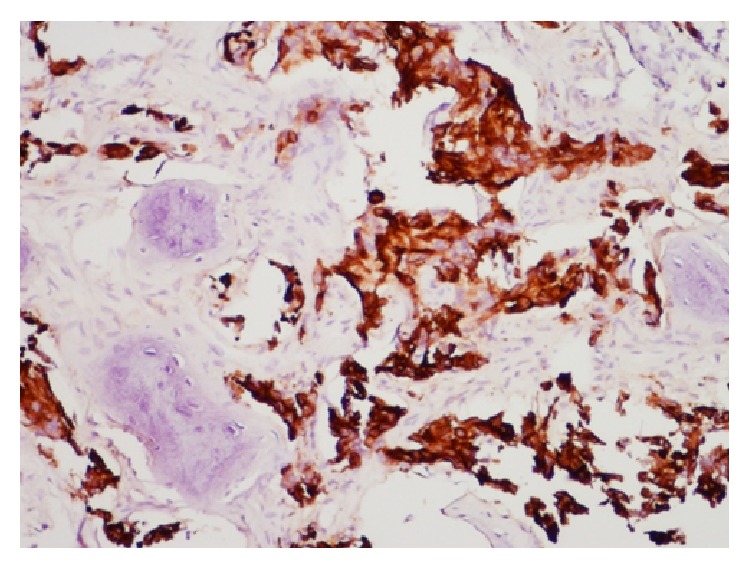
Left petrous bone biopsy, immunohistochemistry staining positive for pan keratin.

**Figure 4:**
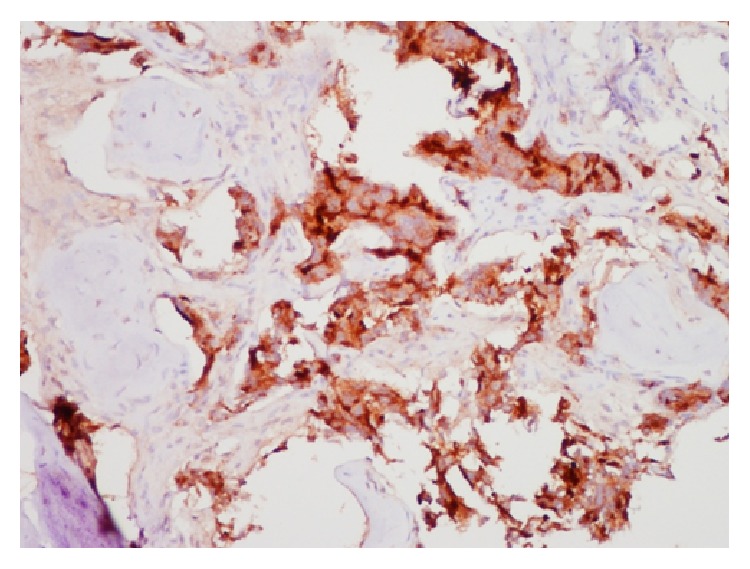
Left petrous bone biopsy, immunohistochemistry staining positive for PSA.

**Figure 5:**
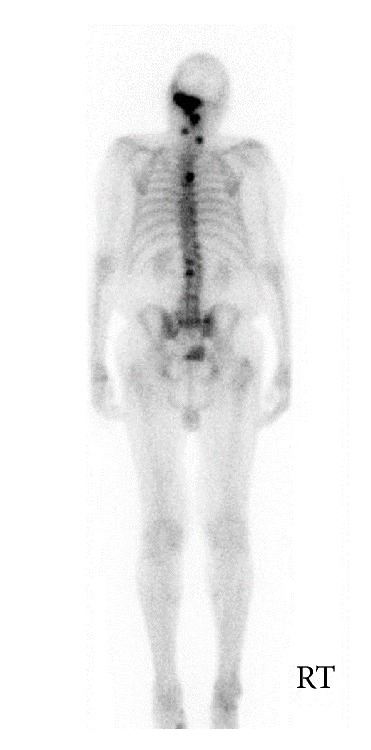
Nuclear medicine bone scan, using technetium 99m-MDP, posterior view; it shows intense radiotracer uptake in the left temporal bone and focal radiotracer localization in the upper thoracic spine.

Biopsy confirmed metastatic prostate carcinoma—the tissues staining for pan keratin (Figure 3) and PSA (Figure 4). To complete a metastatic workup, a technetium 99m scintigraphy was performed, which demonstrated intense radiotracer uptake in the left temporal bone consistent with biopsy-proven metastatic prostate carcinoma (Figure 5). It also showed localization in the upper thoracic spine (T5), which was suspicious for metastasis, as well as uptake in the mid cervical and upper lumbar spine consistent with degenerative changes. The patient has started palliative radiotherapy to the temporal bone and has begun treatment with neoadjuvant Lupron injection (LHRH analog), anti-androgen therapy, and Degarelix. Most recent CT head showed increased interval of metastatic disease. He remains alive at 8 months with no changes in hearing and no recurrence of his left-sided ear pain.

## 3. Discussion

We present the case of a 95-year-old man with history of primary prostate cancer treated 12 years earlier that was seen for new-onset asymmetric hearing loss and otalgia. The tympanic membranes and middle ears were normal; however, based on radiologic findings and eventual biopsy, the patient was diagnosed with extensive metastatic prostate cancer to the left temporal bone. This case (1) demonstrates that a high index of suspicion for unusual etiologies of seemingly benign symptoms must be maintained in elderly patients having prior history of cancer and (2) substantiates the value of temporal bone imaging when diagnosis may be unclear from history and physical exam.

Metastatic cancer involving the temporal bone is often asymptomatic and may be underreported; however, the incidence appears to be rising due to an aging population and better diagnostic modalities that spur improved recognition [13, 19, 21, 22]. Approximately 21 cases of prostate cancer metastases to the temporal bone have been reported in the literature; however, our report is only the 3rd case of metastasis >10 years from the diagnosis and treatment of a primary tumor. Hematogenous spread to the temporal bone is the most common pathway for metastasis [15], but while the petrous apex, mastoid, and internal auditory canal are all possible sites of tumor spread, the petrous apex is the most common site for metastatic seeding [21, 23]. This is likely due to its rich blood supply, provided by Batson's venous plexus [11, 22].

Patients with temporal bone metastases have typically been between their sixth and eighth decade of life. Up to 40% are asymptomatic [9, 14–16]. Patients typically become symptomatic when the mass has grown sufficiently to involve surrounding structures. The most common presentation is hearing loss [11]. Less common otologic and neurologic symptoms are cranial nerve palsies, otalgia, dizziness/vertigo, and tinnitus [14]. Cranial nerves V, VI, and XII are the most common cranial nerves involved and result in facial paresthesia, diplopia, and tongue deviation [24].

The nonspecific features of temporal bone malignancy can make diagnosis difficult; therefore, considering a broad differential diagnosis is important. Possible causes of symptoms that may mimic malignancy are multiple myeloma, chondrosarcoma, chordoma, invasive meningiomas, schwannomas, and petrous apicitis (Table 2) [17, 19, 25]. Making the diagnosis requires imaging and eventual biopsy. Four imaging modalities are commonly employed to aid in the diagnoses of temporal bone tumor: CT, MRI, radionuclide bone scan, and a FDG-PET scan. CT and MRI have the greatest sensitivity and are extremely useful in detecting metastasis [10, 26]. These two modalities compliment each other as the CT shows bony involvement and MRI outlines the soft tissues of the internal auditory canal [17]. The addition of radionuclide bone scan with MRI and/or CT scans increases the overall sensitivity and may improve detection by detecting distant bone metastases and assessing response to therapy [10].

An elevated PSA (prostate specific antigen) in patients with history of primary prostate cancer should increase suspicion for metastatic disease [27]. PSA levels typically rise with metastasis and correlate to tumor volume; however, PSA levels may remain low to normal in patients with early prostate cancer metastasis, as seen in our patient. This reasoning is unclear [10, 28].

Treatment with surgery, radiation, and chemotherapy is aimed at palliation [19]. Surgery is usually required for tissue diagnosis and may be used for debulking and symptomatic patients. Overall, survival in patients with metastatic cancer of the temporal bone is low, with average survival time after diagnosis being <2 years [26]. Patients presenting with cranial nerve palsies typically survive <6 months [10, 26]. New systemic treatment options have become available for metastatic prostate cancer, including hormonal, chemotherapeutic, and immunotherapeutic agents, bone-targeted therapies, and radiopharmaceuticals. Although androgen deprivation therapy remains the first line for treatment for metastatic disease, a standardized sequence of treatment has not yet been developed [29].

## 4. Conclusion

Clinicians must maintain a high index of suspicion for metastatic disease in high-risk patients of advanced age or those having a prior history of malignancy. Early imaging may help prevent a delay in diagnosis. Early diagnosis and treatment is essential to maximize therapeutic responses.

## Figures and Tables

**Table 1:** Reported cases of prostatic metastases to the temporal bone.

Source	Age	Presenting symptom(s)	Prior history of prostatic carcinoma	Radiologic findings of TBM	Histologic findings of TBM	Treatment/follow-up	PSA levels (0–4.0 ng/mL)
Janczewski and Fujital, 1972	77	Generalized bone pain, vertigo, ataxia, left 8th nerve paralysis	Yes (4 yr priorly, hormonal therapy given)	NR	NR	Died of extensive metastatic prostatic carcinoma	NR

Helcl and Malec, 1973	57	Tinnitus, hearing loss, temporomandibular joint pain	No (4 mo later vertebral lesions developed, primary site found on search)	Destruction of apex of right pyramidal bone	NR	Irradiation, hormonal therapy: NR	NR

Applebaum and Dolsky, 1977	64	Ear pain	No (primary site found on search)	Destructive lesion in petrous apex	Poorly differentiated adenocarcinoma	Hormonal therapy, died of tumor after 4 mo	NR

Coppola and Salanga, 1980	50	Left-sided ear pain, preauricular tenderness, hearing loss	Yes (4 yr priorly, well-differentiated treated by TURP)	Erosion of left temporal bone	Poorly differentiated adenocarcinoma	Irradiation, alive with stable disease 1 yr after TBM	NR

Schrimpf et al., 1982	81	Dizziness, right-sided ear pain, hearing loss	No (1 yr later prostate biopsy revealed well-differentiated carcinoma)	Dense sclerosis of mastoid bone, a defect in petrous bone	Moderately differentiated adenocarcinoma	Hormonal therapy, alive with stable disease 1 yr after TBM	NR

Castaldo et al., 1983	67	Left jaw pain and facial weakness	4 years earlier he was admitted to hospital for bladder outlet obstruction	CT scan showed metastatic lesion of the left temporal bone invading the left temporal lobe	NR	3000 rad whole brain radiation	NR

Jung et al., 1986	75	Facial palsy with CN V and XII involvement	NR	NR	Undifferentiated carcinoma of the prostate gland	NR	NR

Svare et al., 1988	NR	CNVIII involvement	NR	Skull X-ray showed right temporal bone lesion	NR	Radiotherapy	NR

Sahin et al., 1991 [30]	69	Dizziness, right-sided temporal pain, hearing loss	Yes (3 yr priorly, stage, irradiation given)	Osteoblastic lesion in the temporal bone with epidural extension	Poorly differentiated adenocarcinoma with immunoreactivity for PAP and PSA	Irradiation, chemotherapy alive with stable disease 6 months after TBM	62.8 ng/mL

Sahin et al., 1991 [30]	73	Right-sided ear pain, tinnitus, hearing loss	No (found on search)	Osteolytic destructive mass in petrous bone with soft tissue component	Moderately differentiated adenocarcinoma with immunoreactivity for PAP and PSA	Hormonal therapy, alive 4 yr after TBM	NR

Pringle et al., 1993	50	Sudden onset left-sided deafness and tinnitus associated with pain in his left ear, around his left eye and radiating into the back of the head	Prostate cancer in 1988 and treated with transurethral resection	MRI revealed a large enhancing lesion on the right side adjacent to the internal auditory meatus	Metastatic prostatic carcinoma	Local radiotherapy and goserelin. Eighteen months after treatment patient was alive	NR

Hellier et al., 1997	60	Two-month history of a progressive loss of function of the left CNVII-XII, pulsatile tinnitus and left-sided deafness	Rectal examination revealed a rock hard smooth prostate compatible with prostatic carcinoma	Vascular mass eroding the intralabyrinthine portion of the temporal bone and extending into the petrous apex	Metastatic prostatic adenocarcinoma	Radiotherapy plus anti-androgen treatment	NR

Messina et al., 1999	75	6-month history of progressive weight loss, initial visual impairment and paraphasia	Yes (15 yr priorly, underwent radical prostatectomy and treated with external-beam radiation therapy)	Extra-axial osteoblastic lesion arising from the left petrous and occipital bones	Immunohistochemical staining positive for prostate-specific acid phosphatase	Temporal, occipital, and parietal bone resection with external-beam radiation and bicalutamide therapy	26 ng/mL

Schwetschenau et al., 2001	60	Right-sided ear pain, vertigo, hearing loss, watery otorrhea, forehead parathesis	Yes (5 yr priorly, treatment not discussed)	CT scan showed sclerotic bones of the anterior canal fossa	Immunohistochemical staining positive for prostate-specific acid phosphatase	Radiation therapy	1200 ng/mL

McAvoy et al., 2002	64	1-day history of binocular horizontal diplopia	1-week history of diagnosed prostate cancer	CT scan showed bony destruction of the right petrous apex and paracavernous region	NR	Hormonal therapy and alive 1 year later	NR

McDermott et al., 2004	68	Facial droop (CNVII)	Metastatic disease present at time of the original diagnosis of prostate carcinoma	MRI showed petrous bone involvement	NR	Treated with a course of external beam radiation therapy	NR

McDermott et al., 2004	68	Facial droop (CNVII)	Yes	MRI showed clivus and temporal bone involvement	NR	Treated with a course of external beam radiation therapy	NR

Malloy, 2007	66	Four-day history of blurred vision that was worse when he looked left and medial deviation of the left eye without pain or other neurologic deficits	Recent diagnoses of prostate cancer for which he was being treated	Two masses were found on MRI. One was 1.6 × 2.4 × 1.8 in size within the mid and left clivus and involving the left cavernous sinus. A secondary mass was found in the left temporal lobe	NR	Radiotherapy and chemotherapy. The patient was alive 2.5 years after initial presentation	NR

Mitchell et al., 2008	55	Progressive onset left-sided facial weakness and occipital and neck pain	No (found on search)	CT showed permeative bone destruction in the left skull base, involving the lower petrous temporal bone	NR	Luteinizing hormone-releasing hormone agonist treatment with anti-androgen cover with palliative radiotherapy	88.9 ng/mL

Alvo et al., 2012 [17]	63	3-month history of headache, right-sided hearing loss, and instability, without vertigo, nausea, or otalgia	11 years priorly the patient was diagnosed with prostate cancer and had undergone prostatectomy plus radiotherapy	CT and MRI showed infiltrative mass in the right petrous apex and clivus, compromising the internal auditory canal	NR	Hormonal therapy with leuprolide and radiotherapy and was stable six months later	63.2 ng/mL

**Table 2:** Imaging characteristics of temporal bone lesions.

	CT scan	MRI, T1 weighted imaging	MRI, T2 weighted imaging	MRI, gadolinium
Schwannoma	Intermediate density	Intermediate (cysts may be low, hemorrhage high)	Intermediate (cysts high, hemorrhage variable)	Avid, homogenous enhancement

Mucocele	Expansile, no bony destruction	Variable, typically low	High	No enhancement

Acute petrous apicitis	Air-fluid levels in air cells without bony destruction	Low	High	Mild

Cholesterol granuloma	Expansile	High	Variable, usually high	No enhancement

Cholesteatoma	Bony erosion, remodeling	Intermediate to low intensity	High	No enhancement

Chondrosarcoma	Bony erosions and mineralized matrix	Intermediate to low intensity	High +/− some heterogeneity if calcified matrix	Avid enhancement

Meningioma	Intermediate density	Intermediate (cysts may be low, hemorrhage high)	Intermediate (cysts high, hemorrhage variable)	Avid, homogeneous enhancement
